# Risk factors, mechanisms, and clinical outcomes of stroke in young adults presenting to a North Central London stroke service: UCL Young Stroke Systematic Evaluation Study (ULYSSES)

**DOI:** 10.1177/23969873251314360

**Published:** 2025-01-23

**Authors:** Raafiah Mussa, Gareth Ambler, Hatice Ozkan, Kitti Thiankhaw, Maryam Aboughdir, Imogen Smedley, John Mitchell, Gargi Banerjee, Hans Rolf Jäger, Alex Leff, Richard Perry, Robert J Simister, Arvind Chandratheva, David J Werring

**Affiliations:** 1 Department of Brain Repair and Rehabilitation, Stroke Research Centre, UCL Queen Square Institute of Neurology, London, UK; 2 Department of Statistical Science, UCL, London, UK; 3 Comprehensive Stroke Service, National Hospital for Neurology and Neurosurgery, UCL Hospitals NHS Foundation Trust, London, UK; 4 Department of Neurology, Royal Free Hospital, Royal Free London NHS Foundation Trust, London, UK; 5 Department of Neurology, North Middlesex University Hospital, North Middlesex University Hospital NHS Trust, London, UK; 6 MRC Prion Unit at UCL, Institute of Prion Diseases, London, UK; 7 Neuroradiological Academic Unit, Department of Brain Repair and Rehabilitation, UCL Queen Square Institute of Neurology, London, UK

**Keywords:** Stroke in young adults, stroke recurrence, functional outcome

## Abstract

**Introduction:**

Stroke incidence in younger adults is increasing worldwide yet few comprehensive studies exist from a UK population. We investigated the risk factors, mechanisms, functional outcome and stroke recurrence rate in a cohort of young adults with stroke.

**Patients and methods:**

We included consecutive patients (<55 years) with ischaemic stroke or intracerebral haemorrhage (ICH) admitted to the University College London Hospitals Hyperacute Stroke Unit between 2017 and 2020. Ischaemic stroke was classified using Trial of Org 10172 in Acute Stroke Treatment (TOAST) criteria and ICH using modified CLAS-ICH criteria. Multivariable logistic regression was performed to identify predictors of unfavourable functional outcome (modified Rankin Scale [mRS] > 1) at 6 months.

**Results:**

Five hundred fifty-two patients were included (median age 47, IQR 41–51; 33% female; 76% ischaemic stroke). Common risk factors included dyslipidaemia (57%), hypertension (40%), and cigarette smoking (34%). Ischaemic stroke was mostly due to cardioembolism (22%). Probable cerebral small vessel disease was the most frequent ICH aetiology (53%). Unfavourable functional outcome was prevalent in 50% at 6 months and was associated with ICH (OR 2.02, 95%CI 1.14–3.58, *p* = 0.017), female sex (OR 1.62, 95%CI 1.03–2.55, *p* = 0.037), admission stroke severity (per point increase, OR 1.11, 95%CI 1.07–1.16, *p* < 0.001) and pre-morbid mRS 2-5 (OR 3.16; 95%CI 1.11–9.03, *p* = 0.032). 4.4% had a recurrent stroke within 6 months.

**Discussion and conclusion:**

Traditional cardiovascular risk factors are common in young adults with stroke. Unfavourable functional outcome is associated with female sex, ICH, severe stroke and pre-morbid disability. These findings can inform national stroke prevention and rehabilitation strategies.

## Graphical abstract

**Graphical abstract:**
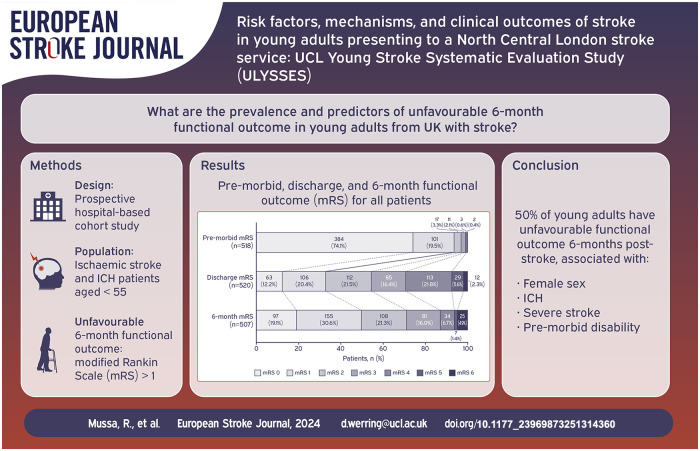

## Introduction

While stroke incidence is decreasing among older adults in high-income countries, it is increasing among younger adults.^1^ In England, between 2001 and 2010, stroke event rates decreased overall, yet there was a 2% annual increase among adults aged 35–54.^2^

Stroke can have a large personal, societal and financial impact on younger people, who are often in their most economically productive and demanding years of employment; in the longer term, there is a higher risk of death compared with the general population.^3,4^ Thus, there is an urgent need to gain a better understanding of the mechanisms and outcomes of stroke and to improve primary and secondary prevention strategies, as well as neurorehabilitation, in this age group. Limitations of previous data include heterogeneity in the definition of young stroke, short follow-up periods (⩽3 months) and few studies including people with intracerebral haemorrhage (ICH).^5,6^

There are few data from the UK investigating detailed baseline characteristics and predictors of unfavourable 6-month functional outcome post-stroke in young adults, particularly concerning sex, ethnicities and socioeconomic status. We therefore established ULYSSES (UCL Young Stroke Systematic Evaluation Study); a cohort study of patients with ischaemic stroke or ICH <55 years old in which we sought to determine the risk factors, mechanisms and clinical outcomes (including functional outcome and stroke recurrence) of stroke in young adults.

## Patients and methods

ULYSSES is a prospective cohort study of young adults presenting to the University College London Hospitals Hyperacute Stroke Unit (UCLH HASU) with acute stroke. UCLH provides specialised stroke care to an ethnically diverse population of approximately 1.6 million people from five North Central London boroughs. Patients were included if they were: (1) <55 years old (consistent with population-based studies from the UK)^7,8^; (2) clinically diagnosed with acute ischaemic stroke or ICH (confirmed on CT or MRI by a consultant neuroradiologist) and (3) admitted between 1st January 2017 and 1st January 2020. Patients who were admitted to and discharged directly from the neurosurgery unit for acute stroke were also identified and included by screening neurosurgery admission records.

Routine clinical data including patient demographics, medical history, admission stroke severity (National Institutes of Health Stroke Scale (NIHSS)) and functional ability (modified Rankin Scale (mRS)) at hospital admission and HASU discharge (approximately 1 week following hospital admission) were obtained from electronic health records (see Table S1 for risk factor definitions). Socioeconomic deprivation was calculated using the Index of Multiple Deprivation (IMD),^9^ a multi-domain measure of relative deprivation derived from aggregated neighbourhood-level data. Patient postcodes were matched to their corresponding neighbourhood to determine whether they lived in a deprived area (see Table S2 for definitions).

Patients received a comprehensive standardised diagnostic workup including brain and neurovascular imaging, cardiac monitoring and laboratory testing (listed in Table S3). At least two raters (RM, KT) determined the mechanism of stroke using the Trial of Org 10172 in Acute Stroke Treatment (TOAST) classification^10^ for ischaemic stroke. The Risk of Paradoxical Embolism (RoPE) score and PASCAL classification were used to define PFO-associated stroke as cardioembolic.^11^ Cases of disagreement were resolved by consensus. ICH was classified as probable cerebral small vessel disease (cSVD); macrovascular; other secondary cause and undetermined aetiology, using a modified CLAS-ICH classification.^12,13^

Follow-up at 6 months was collected as part of routine clinical care by trained practitioners, primarily through outpatient clinic visits and telephone appointments. However, additional support measures including home visits and postal questionnaires were provided to reduce burden on patients with moderate to severe impairments. Functional outcome was measured using the mRS.^14^ Unfavourable functional outcome was defined as mRS >1.^15–18^ Stroke recurrence (defined as a further imaging confirmed ischaemic stroke or ICH within 6 months of HASU discharge) was ascertained using the Questionnaire for Verifying Stroke-Free Status (QVSFS)^19^ and all available electronic health records.

### Data source and ethics statement

ULYSSES is a sub-study of the Stroke Investigation Group in North And central London (SIGNAL) registry. SIGNAL was approved by the UCLH NHS Foundation Trust Governance Review Board as a continuous service evaluation of a comprehensive clinical care programme (5-201920-SE) and the London South-East REC (24/LO/0368); informed patient consent was not required.

### Statistical analysis

Data were analysed using STATA version 18. According to visual histogram, all continuous variables had non-normal distributions and were reported as median (interquartile range (IQR)). To determine factors associated with unfavourable functional outcome, categorical data were compared using the Pearson chi-squared test or Fisher’s exact test and continuous data were compared using the Wilcoxon rank-sum test. Variables with *p*-values <0.2 in univariable analysis were entered into a multivariable logistic regression analysis and thereafter, variables with *p*-values <0.05 were considered statistically significant predictors of unfavourable outcome. We investigated unfavourable functional outcome according to sex by adding interaction terms for sex in the multivariable model. A multivariable logistic regression analysis was not conducted to predict factors associated with stroke recurrence because of the small number of recurrent strokes.

## Results

### Patient characteristics

Five hundred fifty-two patients (median age 47, IQR 41–51; 33% female) were included (see Figure S1 for age distribution). Most of the cohort was of White ethnic background (51%), followed by Other (27%), Black (11%) and Asian (7.8%) ethnicities. Cardiovascular risk factors were common including dyslipidaemia (57%), hypertension (40%), cigarette smoking (34%) and diabetes (14%). Table 1 and Table S4 describe detailed characteristics for patients included in the analysis.

**Table 1.** Patient demographics and clinical characteristics for the total cohort.

Characteristics	*n* = 552
Age (years), median (IQR)	47 (41–51)
Female, *n* (%)	184 (33.3)
Ethnicity, *n* (%)	
White	283 (51.3)
Black	61 (11.0)
Asian	43 (7.8)
Other	147 (26.6)
Unknown	18 (3.3)
Socioeconomic deprivation, median (IQR) (*n* = 530)	4 (3–6)
Medical history, *n* (%)	
Hypertension	219 (39.7)
Diabetes mellitus	78 (14.1)
Dyslipidaemia (*n* = 476)	273 (57.4)
Family history of TIA/stroke	67 (12.1)
Previous TIA/stroke	97 (17.6)
Migraine	52 (9.4)
With aura	11 (2.0)
Without aura	41 (7.4)
Cigarette smoking	186 (33.7)
Recreational drug use	49 (8.9)
Excess alcohol consumption	60 (10.9)
Stroke type, *n* (%)	
Ischaemic stroke	419 (75.9)
Intracerebral haemorrhage	133 (24.1)
Admission NIHSS, median (IQR) (*n* = 489)	4 (2–8)

NIHSS: National Institute of Health Stroke Scale; TIA: transient ischaemic attack; IQR: interquartile range.

Values are presented as median (IQR) for continuous variables and *n* (%) for categorical variables where % represents the proportion of column total. For variables with missing data, number of available records used to calculate the proportion is provided.

**Figure 1.**
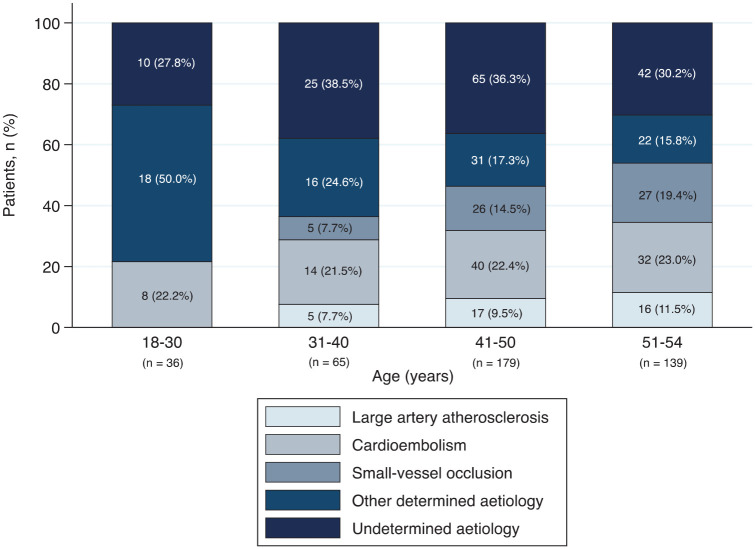
TOAST classification for patients with ischaemic stroke, stratified by age group. TOAST: Trial of Org 10172 in Acute Stroke Treatment. Tables S5 to S7 provide further detailed information on ischaemic stroke classifications.

Ischaemic stroke was most frequently due to cardioembolism (22%), followed by other determined aetiology (21%), small-vessel occlusion (14%) and large artery atherosclerosis (9.1%). Thirty-four percent remained undetermined. Approximately a third of cardioembolic stroke were due to patent foramen ovale (PFO; 32%) and about 20% due to atrial fibrillation. Ischaemic stroke of other determined aetiology was mostly arterial dissection (51%). TOAST aetiology according to age is shown in Figure 1. Tables S5 to S7 provide detailed information on ischaemic stroke classifications.

Twenty-four percent of patients had an ICH, of which the mechanism was probable cSVD in 53%. A macrovascular cause was identified in 18%, of which arteriovenous malformation (AVM) was the most common. Tables S8 to S9 provide detailed information on ICH aetiology.

### Functional outcome

**Figure 2.**
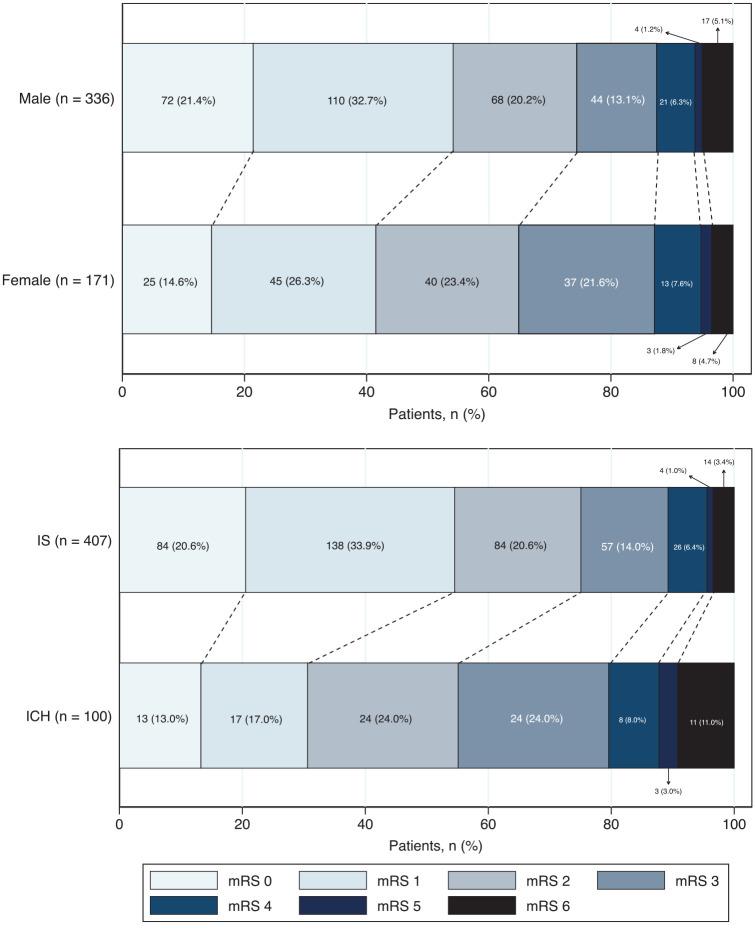
Six-month functional outcome (mRS) according to sex and stroke type. mRS: modified Rankin Scale; IS: ischaemic stroke; ICH: intracerebral haemorrhage.

**Figure 3.**
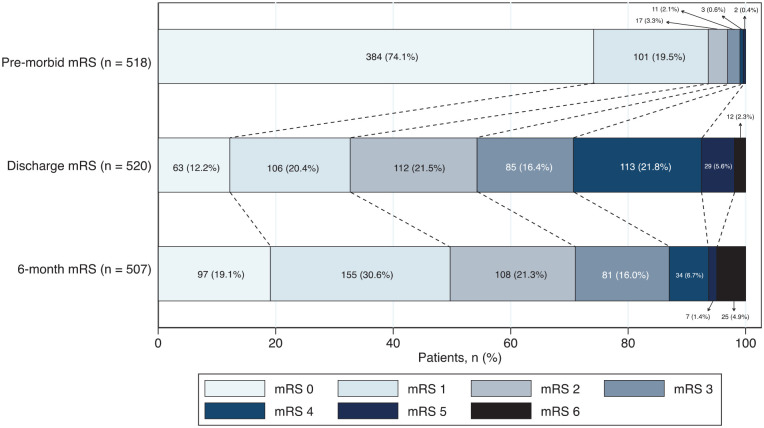
Pre-morbid, discharge, and 6-month functional outcome (mRS) for all patients. mRS: modified Rankin Scale.

Six-month functional outcome was obtained in 92% (30 lost to follow-up; 15 refused to participate; see Figure S4) and was unfavourable (mRS >1) in 50% (95%CI 0.46–0.55); 4.9% (95%CI 0.03–0.07) had died. Over half of females (59%), patients with ICH (70%), and patients of Black (63%) and Other (54%) ethnicities had an unfavourable outcome at 6 months. Figures 2 and 3 show proportions of mRS scores at each time-point for all patients and at 6 months stratified by sex and stroke type. Patient characteristics by sex, stroke type and 6-month follow-up status are shown in Tables S10 to S12.

Several factors independently predicted unfavourable functional outcome in the multivariable logistic regression, including ICH (OR 2.02, 95%CI 1.14–3.58, *p* = 0.017), female sex (OR 1.62, 95%CI 1.03–2.55, *p* = 0.037), admission stroke severity (per point increase, OR 1.11, 95%CI 1.07–1.16, *p* < 0.001) and pre-morbid mRS 2–5 (OR 3.16; 95%CI 1.11–9.03, *p* = 0.032; see Table 2). No significant results were obtained from the interaction model for sex-related differences in functional outcome (*p* = 0.4774; see Figures S2–S3 and Table S11 for patient characteristics stratified by sex).

**Table 2.** Predictors of unfavourable functional outcome (mRS >1) at 6 months (*n* = 450).

Characteristics	OR	95%CI	*p*-Value
Age (per year)	1.024	0.997–1.051	0.085
Sex			
Female	1.620	1.030–2.548	0.037*
Ethnicity			0.164
White	Ref	Ref	Ref
Black	2.064	1.029–4.141	
Asian	0.762	0.341–1.701	
Other	1.209	0.740–1.976	
Unknown	0.473	0.085–2.646	
Socioeconomic deprivation per domain (per decile)			
Income	1.055	0.823–1.352	0.671
Employment	0.947	0.732–1.226	0.682
Health disability	0.980	0.852–1.126	0.772
Living environment	0.914	0.814–1.027	0.131
Medical history			
Diabetes mellitus	1.522	0.842–2.752	0.164
Ischaemic heart disease	1.398	0.638–3.062	0.403
Patent foramen ovale	0.638	0.290–1.404	0.264
Migraine	1.225	0.599–2.505	0.579
Stroke type			
Ischaemic stroke	Ref	Ref	Ref
Intracerebral haemorrhage	2.017	1.136–3.579	0.017*
Admission NIHSS (per point)	1.113	1.068–1.160	<0.001*
Pre-morbid mRS			
No disability (mRS 0–1)	Ref	Ref	Ref
Disability (mRS 2–5)	3.162	1.107–9.032	0.032*

mRS: modified Rankin Scale; NIHSS: National Institute of Health Stroke Scale; TIA: transient ischaemic attack.

Variables with *p*-values <0.2 in univariable analysis (Table S4) were included in the multivariable model. Medication history, inpatient treatment, discharge mRS and stroke recurrence were either omitted from the model or were excluded because they are not applicable to intracerebral haemorrhage patients in the cohort.

* Statistically significant variables (*p* < 0.05).

We conducted additional exploratory analyses on the effects of ethnicity. Using the multivariable logistic regression model described above, with further adjustment for hypertension, we found that Black ethnicity was significantly associated with unfavourable functional outcome compared to both White (OR 2.07, 95%CI 1.02–4.20, *p* = 0.044) and Asian ethnicity (OR 2.71, 95%CI 1.04–7.08, *p* = 0.042) (see Tables S13–S14).

4.4% (95%CI 0.03–0.06) of patients had recurrent ischaemic stroke or ICH within 6 months. A medical history of previous TIA or stroke was a strong predictor (*p* < 0.001) of recurrent stroke in univariable analysis.

## Discussion

This study provides new data on the mechanisms, predictors, and outcomes of stroke in young people from a large ethnically diverse UK population. Important findings are: (1) 50% of young adults with stroke have unfavourable 6-month functional outcome; (2) female sex and ICH are independently associated with unfavourable outcome and (3) Black ethnicity is associated with unfavourable outcome compared to either White or Asian ethnicity.

We observed a higher rate of unfavourable functional outcome (50%) after stroke in younger adults compared to previous studies (18%–39%),^15–17,20^ which mostly investigated outcome at 3 months. Reasons for the differences in unfavourable outcome in our study could include our unselected and ethnically diverse metropolitan population or other differences in case-mix. Most previous studies were in homogeneous populations with a generally lower median age and age cut-off. We found that 70% of ICH patients had an unfavourable 6-month functional outcome compared to 50% of ischaemic stroke patients. This could be explained by ICH patients having higher admission NIHSS scores and greater disability (mRS 2–5) at hospital discharge.

Although many studies use mRS >2 as a measure of unfavourable functional outcome in younger populations, freedom from disability (i.e. mRS 0–1) is perhaps a more appropriate measure because younger adults face societal pressures including work-related and family caregiver obligations. A ‘favourable’ outcome after stroke as a young adult should ideally involve a return to pre-morbid levels of ability; most studies to date have not addressed this issue. Our observations are consistent with recent findings suggesting that younger people with stroke suffer disproportionate rates of unemployment and mental health issues.^21^ This, allied with the increased survival rates across all age groups has important implications for the provision of post-hospital care for younger adults after stroke.^22^

Our finding that females have a 1.6-fold higher risk of unfavourable functional outcome compared to males aligns with population-based studies from Spain and the Netherlands, which reported that females (⩽50 years) have a twofold to threefold higher risk of having an mRS >2 at hospital discharge^23^ and approximately 10 years^24^ after ischaemic stroke compared to males, albeit not in the youngest patients (15–30 years).^23^

Sex differences in stroke outcome could be explained by females: (a) being an average of 4 years older at stroke onset; (b) having worse pre-morbid disability and comorbidities and (c) having a tendency to experience more severe strokes than males.^25^ However, we found an increased risk of unfavourable functional outcome in females despite adjusting for these factors. The effects of sex steroid hormones, sex-specific differences in coagulation, thrombolysis and inflammatory responses to cerebral ischaemia are potentially relevant, given the neuroprotective mechanisms of oestrogen and higher levels of plasminogen activator inhibitor-1 (affecting recanalisation after thrombolysis) and C-reactive protein in females.^26–30^

Our finding that young patients of Black ethnicity have a twofold higher risk of unfavourable functional outcome compared to patients of White or Asian ethnicity (after adjusting for confounding factors) aligns with a systematic review which found that patients of Black ethnicity generally achieve poorer functional outcomes.^31^ Overall, there are limited data on ethnic differences in young adults with stroke and more research is required to evaluate the underlying mechanisms for these disparities.

The most frequent mechanism of ICH was probable cSVD; while previous data indicate that macrovascular ICH is the most frequent cause of ICH in young European cohorts,^5^ our finding could be explained by the higher median age of the current study compared to previous studies including somewhat younger patients (mean age, 38), among whom the ICH aetiology was cSVD-related in 27%–28%.^32,33^

Consistent with previous reports, a large proportion (34%) of ischaemic stroke was undetermined according to the TOAST classification.^34^ This may influence outcomes in young patients as there is limited evidence-based guidance on prevention and treatment in this group. There is a need for improved classification systems specifically tailored to young stroke patients.

Lastly, we observed high proportions of traditional cardiovascular risk factors in this cohort, including dyslipidaemia (57%), hypertension (40%) and cigarette smoking (34%). Previous population-based studies on stroke in young adults have similarly identified these risk factors as the most common.^20,34–37^ Since these factors are modifiable, they should be primary prevention targets in this age group.

This study has several limitations. While our ethnically diverse population is advantageous for exploring the effects of ethnicity on stroke, the results may not be generalisable to less heterogeneous populations. Additionally, due to limitations in our dataset, a large proportion of patients were categorised as belonging to ‘Other’ minority ethnic groups and socioeconomic deprivation was measured at the geographical rather than individual level.^38^ Selection bias is possible as we included only patients admitted to the UCLH HASU and the inclusion of neurosurgical cases of ICH may contribute to the lower proportion of ischaemic stroke in our cohort. However, the consecutive inclusion of young patients with ischaemic stroke and ICH from a broad catchment area helps mitigate this bias and enhances the applicability of our findings to similar urban populations. Although patients underwent a standardised detailed investigation pathway, investigations such as transoesophageal echocardiogram, urine toxicology screening and extended thrombophilia screening (including protein C and S, and antithrombin III) were only performed in select cases. While we achieved a high 6-month follow-up rate (92%), patients without 6-month follow-up had higher admission NIHSS scores suggesting they may have been more severely affected. We made every effort to include all patients by offering home visits and postal questionnaires, however, the missing data likely reflects more severe cases of ICH who were admitted to and discharged directly from the neurosurgery unit for acute stroke care, making them more likely to be lost to follow-up. Lastly, clinical outcome post-stroke was measured using the mRS, which may not accurately describe key aspects of function post-stroke, for example, psychological and non-motor sequelae.^39^

## Conclusion

The ULYSSES study provides new data on the baseline factors associated with unfavourable functional outcome 6-months post-stroke in young adults, including female sex, ICH, severe stroke and pre-morbid disability. Identifying these factors could help tailor care pathways towards patients at highest risk (including access to appropriate rehabilitation assessments). Further research is needed to understand the impact of sex-specific and ethnic differences in functional outcome post-stroke in young adults. The high prevalence of ‘traditional’ modifiable cardiovascular risk factors including dyslipidaemia, hypertension and cigarette smoking in young adults requires targeted public health messaging to ensure younger adults know their risks and how to reduce stroke risk.

## Supplemental Material

sj-docx-1-eso-10.1177_23969873251314360 – Supplemental material for Risk factors, mechanisms, and clinical outcomes of stroke in young adults presenting to a North Central London stroke service: UCL Young Stroke Systematic Evaluation Study (ULYSSES) Supplemental material, sj-docx-1-eso-10.1177_23969873251314360 for Risk factors, mechanisms, and clinical outcomes of stroke in young adults presenting to a North Central London stroke service: UCL Young Stroke Systematic Evaluation Study (ULYSSES) by Raafiah Mussa, Gareth Ambler, Hatice Ozkan, Kitti Thiankhaw, Maryam Aboughdir, Imogen Smedley, John Mitchell, Gargi Banerjee, Hans Rolf Jäger, Alex Leff, Richard Perry, Robert J Simister, Arvind Chandratheva and David J Werring in European Stroke Journal
