# Supplementary material for: Risk factors, mechanisms, and clinical outcomes of stroke in young adults presenting to a North Central London stroke service: UCL Young Stroke Systematic Evaluation Study (ULYSSES)
Source: Eur Stroke J. 2025 Jan 23;10(3):844–52. doi: 10.1177/23969873251314360 (PMC11758433; doi:10.1177/23969873251314360)
Supplement: sj-docx-1-eso-10.1177_23969873251314360 – Supplemental material for Risk factors, mechanisms, and clinical outcomes of stroke in young adults presenting to a North Central London stroke service: UCL Young Stroke Systematic Evaluation Study (ULYSSES) [file sj-docx-1-eso-10.1177_23969873251314360.docx]

**SUPPLEMENTARY MATERIAL**

**Risk factors, mechanisms, and clinical outcomes of stroke in young adults presenting to a North Central London stroke service: UCL Young Stroke Systematic Evaluation Study (ULYSSES)**

Raafiah Mussa, Gareth Ambler, Hatice Ozkan, Kitti Thiankhaw, Maryam Aboughdir, Imogen Smedley, John Mitchell, Gargi Banerjee, Hans Rolf Jӓger, Alex Leff, Richard Perry, Robert J. Simister, Arvind Chandratheva, David J. Werring

**Supplementary Tables**

**Table S1.** Definitions of risk factors

**Table S2.** Definitions of the Index of Multiple Deprivation and its sub-domains

**Table S3.** Standardised diagnostic workup performed during hospital admission and outpatient clinic appointments

**Table S4.** Patient demographics and clinical characteristics according to functional outcome (mRS) at 6 months (n=507)

**Table S5.** TOAST classifications for patients with ischemic stroke according to 6-month mRS outcome

**Table S6.** Sources of cardioembolic stroke according to 6-month mRS outcome

**Table S7.** Causes of other aetiology for ischaemic stroke according to 6-month mRS outcome

**Table S8.** Intracerebral haemorrhage aetiologies according to 6-month mRS outcome

**Table S9.** Macrovascular causes of intracerebral haemorrhage according to 6-month mRS outcome

**Table S10.** Patient demographics and clinical characteristics according to stroke type (n=552)

**Table S11.** Patient demographics and clinical characteristics according to sex (n=552)

**Table S12.** Patient demographics and clinical characteristics according to 6-month follow-up status (n=552)

**Table S13.** Exploratory analysis on unfavourable functional outcome (defined as mRS >1) at 6 months in patients of Black ethnicity compared to patients of White ethnicity (n=450)

**Table S14.** Exploratory analysis on unfavourable functional outcome (defined as mRS >1) at 6 months in patients of Black ethnicity compared to patients of Asian ethnicity (n=450)

**Supplementary Figures**

**Figure S1.** Histogram showing age distribution of the study population (n=552)

**Figure S2.** Histogram showing admission NIHSS distribution for males (n=325)

**Figure S3.** Histogram showing admission NIHSS distribution for females (n=164)

**Figure S4.** Flowchart of patients included in the analysis for 6-month functional outcome (mRS), including methods of ascertainment

**Table S1.** Definitions of risk factors

| Hypertension: | Diagnosed during hospital admission or a history of hypertension according to the 2003 WHO criteria as systolic blood pressure ≥140 mmHg and/or diastolic blood pressure ≥90 mmHg. |
| --- | --- |
| Diabetes mellitus: | Diagnosed during hospital admission or a history of diabetes according to the 1999 WHO criteria as fasting plasma glucose ≥7.0 mmol/L (126 mg/dL). |
| Dyslipidaemia: | Fasting bloods at time of hospital admission show elevated total (≥5.0 mmol/L) or low-density lipoprotein (≥3.0 mmol/L) cholesterol levels, and/or a low high-density lipoprotein (<1.0 mmol/L) cholesterol level. |
| Family history of TIA/stroke: | History of TIA or stroke in a first-degree relative as reported by the patient. |
| Previous TIA/stroke: | History of previous TIA or stroke as reported by the patient. |
| Heart failure: | Left ventricular ejection fraction ≤40% as identified during hospital admission or reported in patients’ medical history. |
| Ischaemic heart disease: | Recent history of ST-segment elevation myocardial infarction or non-ST-segment elevation myocardial infarction. |
| Atrial fibrillation: | As reported in patients’ medical history or as identified during hospital admission on electrocardiogram. |
| Patent foramen ovale: | As reported in patients’ medical history or as identified during hospital admission on transthoracic echocardiogram. |
| Antiphospholipid syndrome: | Positive antiphospholipid antibodies (lupus anticoagulant, anti-β2-glycoprotein 1, and anticardiolipin antibody) at two or more consecutive occasions, at least 12 weeks apart. |
| Migraine: | History of migraine as reported by the patient. |
| Cigarette smoking: | Currently or during the last 5 years, as disclosed by the patient. |
| Recreational drug use: | Current or previous use of cannabis, cocaine, heroin, amphetamines, methamphetamines, or other recreational drugs, as disclosed by the patient or toxicology testing at time of hospital admission. |
| Excess alcohol consumption: | More than clearly moderate drinking (estimated intake of >112g or 140ml) of pure alcohol per week, as disclosed by the patient or judged by treating clinical team. |

TIA, transient ischaemic attack; WHO, World Health Organisation.

**Table S2.** Definitions of the Index of Multiple Deprivation and its sub-domains^9^

| Index of Multiple Deprivation: | Combines information from the seven domains to produce an overall relative measure of deprivation. |
| --- | --- |
| Income: | Measures the proportion of the population experiencing deprivation relating to low income. This includes people that are out-of-work and those that are in work but who have low earnings. |
| Employment: | Measures the proportion of the working age population in an area who are involuntarily excluded from the labour market. This includes people who would like to work but are unable to do so due to unemployment, sickness or disability, or caring responsibilities. |
| Education: | Measures the lack of attainment and skills in the local population. |
| Health disability: | Measures the risk of premature death and the impairment of quality of life through poor physical or mental health. |
| Crime: | Measures the risk of personal and material victimisation at the local level. |
| Barriers to housing and services: | Measures the physical and financial accessibility of housing and local services. This includes ‘geographical barriers’ (i.e., physical proximity of local services) and ‘wider barriers’ (i.e., affordability and homelessness). |
| Living environment: | Measures the quality of the local environment. This includes the ‘indoors’ living environment (i.e., quality of housing) and ‘outdoors’ living environment (i.e., air quality and road traffic accidents). |

**Table S3.** Standardised diagnostic workup performed during hospital admission and outpatient clinic appointments

| Brain and neurovascular imaging: | - CT/CTA - MR/MRA |
| --- | --- |
| Cardiac monitoring: | - Electrocardiogram (ECG) - Transthoracic echocardiogram (TTE) - 72-hour Holter monitor   Where clinically indicated, transoesophageal echocardiogram (TOE) was performed. |
| Routine blood tests: | - Full blood count - Urea and electrolytes - Fasting lipids and glucose |
| Specialist blood tests: | - Erythrocyte sedimentation rate (ESR) - Fasting homocysteine - Vitamin B12 and folate - Anti-human immunodeficiency virus (HIV) and syphilis - Hepatitis B and C - Lupus anticoagulant (LA), antinuclear antibody (ANA), antineutrophil cytoplasmic antibodies (ANCA), anticardiolipin antibody (aCL), and anti-β2-glycoprotein antibody (anti-β2GP)   Where clinically indicated, an extended thrombophilia screen that includes protein C and S, and antithrombin III were performed. |

**Figure S1.** Histogram showing age distribution of the study population (n=552)

**Figure S2.** Histogram showing admission NIHSS distribution for males (n=325)

NIHSS, National Institute of Health Stroke Scale.

**Figure S3.** Histogram showing admission NIHSS distribution for females (n=164)

NIHSS, National Institute of Health Stroke Scale

**Figure S4.** Flowchart of patients included in the analysis for 6-month functional outcome (mRS), including methods of ascertainment

mRS, modified Rankin Scale.

**Table S4.** Patient demographics and clinical characteristics according to functional outcome (mRS) at 6 months (n=507)

|  | **Favourable outcome**  **(mRS 0-1)**  **(n=252)** | **Unfavourable outcome**  **(mRS 2-6)**  **(n=255)** | ***p-*value** |
| --- | --- | --- | --- |
| **Age (years), median (IQR)** | 47 (38-52) | 48 (43-51) | 0.081 |
| **Female, n (%)** | 70 (27.8) | 101 (39.6) | ***0.005** |
| **Ethnicity, n (%)** |  |  | 0.078 |
| White | 139 (55.2) | 121 (47.5) |  |
| Black | 22 (8.7) | 38 (14.9) |  |
| Asian | 22 (8.7) | 14 (5.5) |  |
| Other | 65 (25.8) | 77 (30.2) |  |
| Unknown | 4 (1.6) | 5 (2.0) |  |
| **Socioeconomic deprivation, median (IQR) *(n=487)** | 4 (3-6) | 4 (2-6) | 0.221 |
| **Socioeconomic deprivation per domain,**  **median (IQR) *(n=487)** |  |  |  |
| Income | 4 (2-6) | 3 (2-6) | 0.157 |
| Employment | 5 (3-7) | 4 (3-7) | 0.118 |
| Education | 7 (4-8) | 6.5 (4-9) | 0.627 |
| Health disability | 6 (5-9) | 6 (5-8) | 0.199 |
| Crime | 4 (2-6) | 4 (2-5) | 0.211 |
| Barriers to housing and services | 2 (1-4) | 2.5 (1-4) | 0.551 |
| Living environment | 3 (2-5) | 3 (2-4) | ***0.034** |
| **Medical history, n (%)** |  |  |  |
| Hypertension | 93 (36.9) | 107 (42.0) | 0.244 |
| Diabetes mellitus | 31 (12.3) | 43 (16.9) | 0.146 |
| Dyslipidaemia *(n=456) | 137 (57.6) | 126 (57.8) | 0.960 |
| Family history of TIA/stroke | 37 (14.7) | 29 (11.4) | 0.268 |
| Previous TIA/stroke | 44 (17.5) | 49 (19.2) | 0.610 |
| Heart failure | 4 (1.6) | 5 (2.0) | 0.750 |
| Ischaemic heart disease | 15 (6.0) | 25 (9.8) | 0.108 |
| Atrial fibrillation | 13 (5.2) | 14 (5.5) | 0.868 |
| Patent foramen ovale | 31 (12.3) | 12 (4.7) | ***0.002** |
| Antiphospholipid syndrome | 8 (3.2) | 9 (3.5) | 0.824 |
| Migraine | 31 (12.3) | 20 (7.8) | 0.095 |
| With aura | 8 (3.2) | 3 (1.2) | 0.123 |
| Without aura | 23 (9.1) | 17 (6.7) | 0.304 |
| Cigarette smoking | 93 (36.9) | 83 (32.6) | 0.303 |
| Recreational drug use | 25 (9.9) | 20 (7.8) | 0.411 |
| Excess alcohol consumption | 31 (12.3) | 28 (11.0) | 0.643 |
| **Stroke type, n (%)** |  |  | ***<0.001** |
| Ischaemic stroke | 223 (88.1) | 185 (72.6) |  |
| Intracerebral haemorrhage | 30 (11.9) | 70 (27.5) |  |
| **Inpatient treatment, n (%) *(n=407)** |  |  |  |
| Intravenous thrombolysis | 37 (16.7) | 46 (24.9) | ***0.041** |
| Mechanical thrombectomy | 7 (3.2) | 20 (10.8) | ***0.002** |
| **Medication history, n (%)** |  |  |  |
| Antiplatelet | 200 (79.4) | 161 (63.1) | ***<0.001** |
| Anticoagulant | 43 (17.1) | 64 (25.1) | ***0.027** |
| Antihypertensive | 103 (40.9) | 144 (56.5) | ***<0.001** |
| Statin | 187 (74.2) | 164 (64.3) | ***0.016** |
| **Admission NIHSS, median (IQR)**  ***(n=470)** | 3 (1-5) | 5 (2-12.5) | ***<0.001** |
| **Pre-morbid mRS, n (%) *(n=503)** |  |  | ***<0.001** |
| Disability (mRS 2-5) | 5 (2.0) | 26 (10.4) |  |
| **Discharge mRS, n (%) *(n=505)** |  |  | ***<0.001** |
| Disability (mRS 2-5) | 120 (47.6) | 204 (80.6) |  |
| **Dead at discharge, n (%)** | … | 12 (4.7) | ***<0.001** |
| **Stroke recurrence at 6 months, n (%)** | 8 (3.2) | 15 (5.9) | 0.143 |

Values are presented as median (IQR) for continuous variables and n (%) for categorical variables, with % representing the proportion of column total. Categorical variables were compared using the Pearson chi-squared test or Fisher’s exact test as appropriate, and continuous variables were compared using the Wilcoxon rank-sum test. * denotes statistically significant variables (*p*<0.05). For those variables with missing data, the number of records available and used to calculate the proportion is provided. mRS, modified Rankin Scale; NIHSS, National Institute of Health Stroke Scale; TIA, transient ischaemic attack; IQR, interquartile range.

**Table S5.** TOAST classifications for patients with ischemic stroke according to 6-month mRS outcome

|  | **Patients with ischaemic stroke**  **(n=419)** | **Proportion with 6-month mRS data available**  **(n=407)** | |  |
| --- | --- | --- | --- | --- |
|  |  | **Favourable outcome**  **(mRS 0-1)**  **(n=222)** | **Unfavourable outcome**  **(mRS 2-6)**  **(n=185)** | ***p-*value** |
| **TOAST classification** |  |  |  | 0.410 |
| Large artery atherosclerosis | 38 (9.1) | 17 (7.7) | 21 (11.4) |  |
| Cardioembolism | 94 (22.4) | 51 (23.0) | 38 (20.5) |  |
| Small-vessel occlusion | 58 (13.8) | 35 (15.8) | 22 (11.9) |  |
| Other aetiology | 87 (20.8) | 49 (22.1) | 36 (19.5) |  |
| Undetermined aetiology | 142 (33.9) | 70 (31.5) | 68 (36.8) |  |

mRS, modified Rankin Scale; TOAST, Trial of Org 10172 in Acute Stroke Treatment.

**Table S6.** Sources of cardioembolic stroke according to 6-month mRS outcome

|  | **Patients with cardioembolic stroke**  **(n=94)** | **Proportion with 6-month mRS data available**  **(n=89)** | |  | **Comments** |
| --- | --- | --- | --- | --- | --- |
|  |  | **Favourable outcome**  **(mRS 0-1)**  **(n=51)** | **Unfavourable outcome**  **(mRS 2-6)**  **(n=38)** | ***p-*value** |  |
|  |  |  |  | 0.157 |  |
| AF | 17 (18.1) | 8 (15.7) | 8 (21.1) |  |  |
| PFO | 30 (31.9) | 22 (43.1) | 7 (18.4) |  |  |
| Other source | 25 (26.6) | 11 (21.6) | 12 (13.6) |  | Impaired LV function (1); mitral valve regurgitation (1); atrial flutter (2); recent MI (2); paroxysmal AF (1); isolated tachycardia (1); Lambl’s excrescences (1); atrial septal defect (1); cardiomyopathy (4); heart failure (2); infective endocarditis (2); rheumatic valve disease (5);  LV thrombus (2) |
| More than one potential source | 8 (8.5) | 3 (5.9) | 5 (13.2) |  | Cardiomyopathy and AF (2); heart failure and LV thrombus (2); rheumatic valve disease and AF (1); heart failure and AF (2); heart failure and cardiomyopathy (1) |
| Undetermined source | 14 (14.9) | 7 (13.7) | 6 (15.8) |  |  |

AF, atrial fibrillation; PFO, patent foramen ovale; LV, left ventricle; MI, myocardial infarction; mRS, modified Rankin Scale.

**Table S7.** Causes of other aetiology for ischaemic stroke according to 6-month mRS outcome

|  | **Patients with stroke of other aetiology**  **(n=87)** | **Proportion with 6-month mRS data available**  **(n=85)** | |  | **Comments** |
| --- | --- | --- | --- | --- | --- |
|  |  | **Favourable outcome**  **(mRS 0-1)**  **(n=49)** | **Unfavourable outcome**  **(mRS 2-6)**  **(n=36)** | ***p-*value** |  |
|  |  |  |  | 0.353 |  |
| Haematological conditions | 25 (28.7) | 11 (22.5) | 12 (33.3) |  | Malignancy (6); protein C deficiency (1); protein S deficiency (1); anti-phospholipid syndrome (12); thrombophilia (3); myeloproliferative disorder (2) |
| Inflammatory cause | 6 (6.9) | 4 (8.2) | 2 (5.6) |  | Intracranial vasculitis (5); Takayasu’s arteritis (1) |
| Genetic cause | 1 (1.2) | 1 (2.0) | … |  | Sickle cell disease (1) |
| Arterial dissection | 44 (50.6) | 29 (59.2) | 15 (41.7) |  | Vertebral (unilateral) (24);  vertebral (bilateral) (1);  internal carotid (unilateral) (18); internal carotid (bilateral) (1). |
| Infective disease | 2 (2.3) | … | 2 (5.6) |  | Neurosyphilis in HIV (2) |
| Drug-associated | 6 (6.9) | 3 (6.1) | 3 (8.3) |  |  |
| Other | 3 (3.5) | 1 (2.0) | 2 (5.6) |  | Cardiac amyloidosis (1); radiation-induced vasculopathy (2) |

HIV, human immunodeficiency virus; mRS, modified Rankin Scale.

**Table S8.** Intracerebral haemorrhage aetiologies according to 6-month mRS outcome

|  | **Patients with ICH**  **(n=133)** | **Proportion with 6-month mRS data available**  **(n=100)** | |  | **Comments** |
| --- | --- | --- | --- | --- | --- |
|  |  | **Favourable outcome**  **(mRS 0-1)**  **(n=30)** | **Unfavourable outcome**  **(mRS 2-6)**  **(n=70)** | ***p-*value** |  |
| **ICH aetiology** |  |  |  | 0.967 |  |
| Probable cerebral small vessel disease | 71 (53.4) | 19 (63.3) | 45 (64.3) |  |  |
| Macrovascular | 24 (18.1) | 4 (13.3) | 7 (10.0) |  |  |
| Other secondary cause | 14 (10.5) | 2 (6.7) | 5 (7.1) |  | Malignancy (7); post-operative (2); moyamoya disease (2); pregnancy-related (1); infective endocarditis (1); drug-associated (1) |
| Undetermined aetiology | 24 (18.1) | 5 (16.7) | 13 (18.6) |  |  |

mRS, modified Rankin Scale; ICH, intracerebral haemorrhage.

**Table S9.** Macrovascular causes of intracerebral haemorrhage according to 6-month mRS outcome

|  | **Patients with macrovascular ICH**  **(n=24)** | **Proportion with 6-month mRS data available**  **(n=11)** | |  |
| --- | --- | --- | --- | --- |
|  |  | **Favourable outcome**  **(mRS 0-1)**  **(n=4)** | **Unfavourable outcome**  **(mRS 2-6)**  **(n=7)** | ***p-*value** |
|  |  |  |  | 0.735 |
| Arteriovenous malformation | 13 (54.2) | 2 (50.0) | 2 (28.6) |  |
| Cavernoma | 6 (25.0) | 1 (25.0) | 3 (42.9) |  |
| Dural arteriovenous fistula | 4 (16.7) | 1 (25.0) | 1 (14.3) |  |
| Venous thrombosis | 1 (4.2) | … | 1 (14.3) |  |

mRS, modified Rankin Scale; ICH, intracerebral haemorrhage.

**Table S10.** Patient demographics and clinical characteristics according to stroke type (n=552)

|  | **Ischaemic stroke**  **(n=419)** | **Intracerebral haemorrhage**  **(n=133)** | ***p-*value** |
| --- | --- | --- | --- |
| **Age (years), median (IQR)** | 48 (41-52) | 47 (42-51) | 0.512 |
| **Female, n (%)** | 143 (34.1) | 41 (30.8) | 0.482 |
| **Ethnicity, n (%)** |  |  | ***<0.001** |
| White | 230 (54.9) | 53 (39.9) |  |
| Black | 46 (11.0) | 15 (11.3) |  |
| Asian | 32 (7.6) | 11 (8.3) |  |
| Other | 105 (25.1) | 42 (31.6) |  |
| Unknown | 6 (1.4) | 12 (9.0) |  |
| **Socioeconomic deprivation, median (IQR) *(n=530)** | 4 (3-6) | 4 (3-6) | 0.619 |
| **Socioeconomic deprivation per domain,**  **median (IQR) *(n=530)** |  |  |  |
| Income | 4 (2-6) | 4 (2-6) | 0.968 |
| Employment | 5 (3-7) | 5 (3-7) | 0.670 |
| Education | 7 (4-8.5) | 6 (4-8) | 0.321 |
| Health disability | 6 (5-9) | 7 (5-9) | 0.722 |
| Crime | 4 (2-6) | 4 (2-6) | 0.619 |
| Barriers to housing and services | 2 (1-4) | 2 (1-4) | 0.554 |
| Living environment | 3 (2-4) | 3 (2-5) | 0.287 |
| **Medical history, n (%)** |  |  |  |
| Hypertension | 143 (34.1) | 76 (57.1) | ***<0.001** |
| Diabetes mellitus | 68 (16.2) | 10 (7.5) | ***0.012** |
| Dyslipidaemia *(n=476) | 220 (56.9) | 53 (59.6) | 0.642 |
| Family history of TIA/stroke | 59 (14.1) | 8 (6.0) | ***0.013** |
| Previous TIA/stroke | 82 (19.6) | 15 (11.3) | ***0.029** |
| Heart failure | 9 (2.2) | … | 0.088 |
| Ischaemic heart disease | 36 (8.6) | 5 (3.8) | 0.064 |
| Atrial fibrillation | 27 (6.4) | 1 (0.8) | ***0.009** |
| Patent foramen ovale | 45 (10.7) | … | ***<0.001** |
| Antiphospholipid syndrome | 17 (4.1) | … | ***0.018** |
| Migraine | 47 (11.2) | 5 (3.8) | ***0.010** |
| With aura | 11 (2.6) | … | 0.059 |
| Without aura | 36 (8.6) | 5 (3.8) | 0.064 |
| Cigarette smoking | 161 (38.4) | 25 (18.8) | ***<0.001** |
| Recreational drug use | 38 (9.1) | 11 (8.3) | 0.778 |
| Excess alcohol consumption | 48 (11.5) | 12 (9.0) | 0.432 |
| **Inpatient treatment, n (%)** |  |  |  |
| Intravenous thrombolysis | 86 (20.5) | … | … |
| Mechanical thrombectomy | 27 (6.4) | … | … |
| **Medication history, n (%)** |  |  |  |
| Antiplatelet | 364 (86.9) | 7 (5.3) | ***<0.001** |
| Anticoagulant | 99 (23.6) | 16 (12.0) | ***0.004** |
| Antihypertensive | 166 (39.6) | 104 (78.2) | ***<0.001** |
| Statin | 339 (80.9) | 21 (15.8) | ***<0.001** |
| **Admission NIHSS, median (IQR)**  ***(n=489)** | 3 (2-7) | 6 (2-15) | ***<0.001** |
| **Pre-morbid mRS, n (%) *(n=518)** |  |  | 0.091 |
| Disability (mRS 2-5) | 23 (5.5) | 10 (10.1) |  |
| **Discharge mRS, n (%) *(n=520)** |  |  | ***<0.001** |
| Disability (mRS 2-5) | 263 (62.8) | 76 (75.3) |  |
| **Dead at discharge, n (%)** | 4 (1.0) | 8 (7.9) | ***<0.001** |
| **Stroke recurrence at 6 months, n (%)** | 17 (4.1) | 7 (5.3) | 0.552 |
| **6-month mRS, n (%) *(n=507)** |  |  | ***<0.001** |
| Unfavourable (mRS 2-6) | 185 (45.5) | 70 (70.0) |  |

Values are presented as median (IQR) for continuous variables and n (%) for categorical variables, with % representing the proportion of column total. Categorical variables were compared using the Pearson chi-squared test or Fisher’s exact test as appropriate, and continuous variables were compared using the Wilcoxon rank-sum test. * denotes statistically significant variables (*p*<0.05). For those variables with missing data, the number of records available and used to calculate the proportion is provided. mRS, modified Rankin Scale; NIHSS, National Institute of Health Stroke Scale; TIA, transient ischaemic attack; IQR, interquartile range.

**Table S11.** Patient demographics and clinical characteristics according to sex (n=552)

|  | **Male**  **(n=368)** | **Female**  **(n=184)** | ***p-*value** |
| --- | --- | --- | --- |
| **Age (years), median (IQR)** | 48 (41-52) | 47 (41-51) | 0.212 |
| **Ethnicity, n (%)** |  |  | ***0.029** |
| White | 199 (54.1) | 84 (45.7) |  |
| Black | 31 (8.4) | 30 (16.3) |  |
| Asian | 26 (7.1) | 17 (9.2) |  |
| Other | 102 (27.7) | 45 (24.5) |  |
| Unknown | 10 (2.7) | 8 (4.4) |  |
| **Socioeconomic deprivation, median (IQR) *(n=530)** | 4 (3-6) | 4 (2-6) | 0.180 |
| **Socioeconomic deprivation per domain,**  **median (IQR) *(n=530)** |  |  |  |
| Income | 4 (2-6) | 4 (2-6) | 0.173 |
| Employment | 5 (3-7) | 4 (3-7) | 0.258 |
| Education | 7 (4-9) | 6 (4-8) | 0.095 |
| Health disability | 6 (5-9) | 6 (5-9) | 0.863 |
| Crime | 4 (2-6) | 4 (2-6) | 0.999 |
| Barriers to housing and services | 3 (1-4) | 2 (1-4) | 0.191 |
| Living environment | 3 (2-5) | 3 (2-4) | 0.169 |
| **Medical history, n (%)** |  |  |  |
| Hypertension | 147 (40.0) | 72 (39.1) | 0.854 |
| Diabetes mellitus | 51 (13.9) | 27 (14.7) | 0.795 |
| Dyslipidaemia *(n=476) | 181 (57.5) | 92 (57.1) | 0.947 |
| Family history of TIA/stroke | 41 (11.1) | 26 (14.1) | 0.311 |
| Previous TIA/stroke | 56 (15.2) | 41 (22.3) | ***0.040** |
| Heart failure | 6 (1.6) | 3 (1.6) | 1.000 |
| Ischaemic heart disease | 29 (7.9) | 12 (6.5) | 0.566 |
| Atrial fibrillation | 17 (4.6) | 11 (6.0) | 0.493 |
| Patent foramen ovale | 31 (8.4) | 14 (7.6) | 0.741 |
| Antiphospholipid syndrome | 8 (2.2) | 9 (4.9) | 0.082 |
| Migraine | 23 (6.3) | 29 (15.8) | ***<0.001** |
| With aura | 6 (1.6) | 5 (2.7) | **0.389** |
| Without aura | 17 (4.6) | 24 (13.0) | ***<0.001** |
| Cigarette smoking | 141 (38.3) | 45 (24.5) | ***0.001** |
| Recreational drug use | 38 (10.3) | 11 (6.0) | 0.090 |
| Excess alcohol consumption | 48 (13.0) | 12 (6.5) | ***0.020** |
| **Stroke type, n (%)** |  |  | 0.482 |
| Ischaemic stroke | 276 (75.0) | 143 (77.7) |  |
| Intracerebral haemorrhage | 92 (25.0) | 41 (22.3) |  |
| **Inpatient treatment, n (%) *(n=419)** |  |  |  |
| Intravenous thrombolysis | 58 (21.0) | 28 (19.6) | 0.730 |
| Mechanical thrombectomy | 15 (5.4) | 12 (8.4) | 0.242 |
| **Medication history, n (%)** |  |  |  |
| Antiplatelet | 248 (67.4) | 123 (66.9) | 0.898 |
| Anticoagulant | 78 (21.2) | 37 (20.1) | 0.767 |
| Antihypertensive | 187 (50.8) | 83 (45.1) | 0.206 |
| Statin | 245 (66.6) | 115 (62.5) | 0.343 |
| **Admission NIHSS, median (IQR)**  ***(n=489)** | 4 (2-8) | 4 (2-9) | 0.437 |
| **Pre-morbid mRS, n (%) *(n=518)** |  |  | ***0.008** |
| Disability (mRS 2-5) | 15 (4.4) | 18 (10.4) |  |
| **Discharge mRS, n (%) *(n=520)** |  |  | 0.191 |
| Disability (mRS 2-5) | 219 (63.5) | 120 (68.6) |  |
| **Dead at discharge, n (%)** | 7 (2.0) | 5 (2.9) | 0.552 |
| **Stroke recurrence at 6 months, n (%)** | 16 (4.4) | 8 (4.4) | 1.000 |
| **6-month mRS, n (%) *(n=507)** |  |  | ***0.005** |
| Unfavourable (mRS 2-6) | 154 (45.8) | 101 (59.1) |  |

Values are presented as median (IQR) for continuous variables and n (%) for categorical variables, with % representing the proportion of column total. Categorical variables were compared using the Pearson chi-squared test or Fisher’s exact test as appropriate, and continuous variables were compared using the Wilcoxon rank-sum test. * denotes statistically significant variables (*p*<0.05). For those variables with missing data, the number of records available and used to calculate the proportion is provided. mRS, modified Rankin Scale; NIHSS, National Institute of Health Stroke Scale; TIA, transient ischaemic attack; IQR, interquartile range.

**Table S12.** Patient demographics and clinical characteristics according to 6-month follow-up status (n=552)

|  | **Patients with 6-month mRS**  **(n=507)** | **Patients without 6-month mRS**  **(n=45)** | ***p-*value** |
| --- | --- | --- | --- |
| **Age (years), median (IQR)** | 47 (41-51) | 47 (41-50) | 0.318 |
| **Female, n (%)** | 171 (33.7) | 13 (28.9) | 0.509 |
| **Ethnicity, n (%)** |  |  | ***<0.001** |
| White | 260 (51.3) | 23 (51.1) |  |
| Black | 60 (11.8) | 1 (2.2) |  |
| Asian | 36 (7.1) | 7 (15.6) |  |
| Other | 142 (28.0) | 5 (11.1) |  |
| Unknown | 9 (1.8) | 9 (20.0) |  |
| **Socioeconomic deprivation, median (IQR) *(n=530)** | 4 (3-6) | 5 (4-7) | ***0.019** |
| **Socioeconomic deprivation per domain,**  **median (IQR) *(n=530)** |  |  |  |
| Income | 4 (2-6) | 5 (3-7) | ***0.024** |
| Employment | 5 (3-7) | 6 (4-8) | 0.051 |
| Education | 7 (4-8) | 7 (4-9) | 0.579 |
| Health disability | 6 (5-9) | 7 (5-9) | 0.241 |
| Crime | 4 (2-6) | 5 (3-7) | ***0.004** |
| Barriers to housing and services | 2 (1-4) | 3 (1-4) | 0.375 |
| Living environment | 3 (2-4) | 4 (3-7) | ***0.010** |
| **Medical history, n (%)** |  |  |  |
| Hypertension | 200 (39.5) | 19 (42.2) | 0.715 |
| Diabetes mellitus | 74 (14.6) | 4 (8.9) | 0.292 |
| Dyslipidaemia *(n=476) | 263 (57.7) | 10 (50.0) | 0.497 |
| Family history of TIA/stroke | 66 (13.0) | 1 (2.2) | ***0.034** |
| Previous TIA/stroke | 93 (18.3) | 4 (8.9) | 0.110 |
| Heart failure | 9 (1.8) | … | 0.368 |
| Ischaemic heart disease | 40 (7.9) | 1 (2.2) | 0.165 |
| Atrial fibrillation | 27 (5.3) | 1 (2.2) | 0.363 |
| Patent foramen ovale | 43 (8.5) | 2 (4.4) | 0.343 |
| Antiphospholipid syndrome | 17 (3.4) | … | 0.212 |
| Migraine | 51 (10.1) | 1 (2.2) | 0.085 |
| With aura | 11 (2.2) | … | 0.318 |
| Without aura | 40 (7.9) | 1 (2.2) | 0.165 |
| Cigarette smoking | 176 (34.7) | 10 (22.2) | 0.089 |
| Recreational drug use | 45 (8.9) | 4 (8.9) | 0.998 |
| Excess alcohol consumption | 59 (11.6) | 1 (2.2) | 0.052 |
| **Stroke type, n (%)** |  |  | ***<0.001** |
| Ischaemic stroke | 407 (80.3) | 12 (26.7) |  |
| Intracerebral haemorrhage | 100 (19.7) | 33 (73.3) |  |
| **Inpatient treatment, n (%) *(n=419)** |  |  |  |
| Intravenous thrombolysis | 83 (20.4) | 3 (25.0) | 0.697 |
| Mechanical thrombectomy | 27 (6.6) | … | 0.356 |
| **Medication history, n (%)** |  |  |  |
| Antiplatelet | 361 (71.2) | 10 (22.2) | ***<0.001** |
| Anticoagulant | 107 (21.1) | 8 (17.8) | 0.598 |
| Antihypertensive | 247 (48.7) | 23 (51.1) | 0.758 |
| Statin | 351 (69.2) | 9 (20.0) | ***<0.001** |
| **Admission NIHSS, median (IQR) *(n=489)** | 3 (2-8) | 6 (4-12) | ***0.014** |
| **Pre-morbid mRS, n (%) *(n=518)** |  |  | 0.263 |
| Disability (mRS 2-5) | 31 (6.2) | 2 (13.3) |  |
| **Discharge mRS, n (%) *(n=520)** |  |  | ***0.016** |
| Disability (mRS 2-5) | 324 (64.2) | 15 (100.0) |  |
| **Dead at discharge, n (%)** | 12 (2.4) | … | 0.546 |
| **Stroke recurrence at 6 months, n (%)** | 23 (4.5) | 1 (2.2) | 0.466 |
| **6-month mRS, n (%) *(n=507)** |  |  | … |
| Unfavourable (mRS 2-6) | 255 (50.3) | … |  |

Values are presented as median (IQR) for continuous variables and n (%) for categorical variables, with % representing the proportion of column total. Categorical variables were compared using the Pearson chi-squared test or Fisher’s exact test as appropriate, and continuous variables were compared using the Wilcoxon rank-sum test. * denotes statistically significant variables (*p*<0.05). For those variables with missing data, the number of records available and used to calculate the proportion is provided. mRS, modified Rankin Scale; NIHSS, National Institute of Health Stroke Scale; TIA, transient ischaemic attack; IQR, interquartile range.

**Table S13.** Exploratory analysis on unfavourable functional outcome (defined as mRS >1) at 6 months in patients of Black ethnicity compared to patients of White ethnicity (n=450)

|  | OR | 95%CI | *p*-value |
| --- | --- | --- | --- |
| Age (per year) | 1.024 | 0.996 – 1.053 | 0.099 |
| Sex |  |  |  |
| Female | 1.619 | 1.028 – 2.550 | ***0.038** |
| Ethnicity |  |  |  |
| White | Ref | Ref | Ref |
| Black | 2.070 | 1.019 – 4.204 | ***0.044** |
| Asian | 0.763 | 0.340 – 1.715 | 0.513 |
| Other | 1.210 | 0.738 – 1.984 | 0.449 |
| Unknown | 0.474 | 0.085 – 2.657 | 0.396 |
| Socioeconomic deprivation per domain (per decile) | |  |  |
| Income | 1.055 | 0.823 – 1.353 | 0.674 |
| Employment | 0.948 | 0.732 – 1.227 | 0.684 |
| Health disability | 0.980 | 0.852 – 1.126 | 0.773 |
| Living environment | 0.914 | 0.814 – 1.027 | 0.131 |
| Medical history |  |  |  |
| Diabetes mellitus | 1.523 | 0.835 – 2.790 | 0.170 |
| Ischaemic heart disease | 1.398 | 0.638 – 3.062 | 0.403 |
| Patent foramen ovale | 0.636 | 0.287 – 1.409 | 0.265 |
| Migraine | 1.227 | 0.597 – 2.521 | 0.578 |
| Hypertension | 0.990 | 0.606 – 1.617 | 0.968 |
| Stroke type |  |  |  |
| Ischaemic stroke | Ref | Ref | Ref |
| Intracerebral haemorrhage | 2.023 | 1.118 – 3.660 | ***0.020** |
| Admission NIHSS (per point) | 1.113 | 1.067 – 1.160 | ***<0.001** |
| Pre-morbid mRS |  |  |  |
| No disability (mRS 0-1) | Ref | Ref | Ref |
| Disability (mRS 2-5) | 3.171 | 1.100 – 9.140 | ***0.033** |

Variables with p-values <0.2 in the univariable analysis (Supplementary Table S4) were included in the multivariable model. Medication history, inpatient treatment, discharge mRS, and stroke recurrence were either omitted from the model or were excluded because they are not applicable to intracerebral haemorrhage patients in the cohort. Hypertension was included in the model as a potential confounding factor. * denotes statistically significant variables (*p*<0.05). mRS, modified Rankin Scale; NIHSS, National Institute of Health Stroke Scale; TIA, transient ischaemic attack; IQR, interquartile range.

**Table S14.** Exploratory analysis on unfavourable functional outcome (defined as mRS >1) at 6 months in patients of Black ethnicity compared to patients of Asian ethnicity (n=450)

|  | OR | 95%CI | *p*-value |
| --- | --- | --- | --- |
| Age (per year) | 1.024 | 0.996 – 1.053 | 0.099 |
| Sex |  |  |  |
| Female | 1.619 | 1.028 – 2.550 | ***0.038** |
| Ethnicity |  |  |  |
| White | 1.310 | 0.583 – 2.942 | 0.513 |
| Black | 2.711 | 1.038 – 7.082 | ***0.042** |
| Asian | Ref | Ref | Ref |
| Other | 1.585 | 0.686 – 3.662 | 0.281 |
| Unknown | 0.621 | 0.097 – 3.985 | 0.615 |
| Socioeconomic deprivation per domain (per decile) | |  |  |
| Income | 1.055 | 0.823 – 1.353 | 0.674 |
| Employment | 0.948 | 0.732 – 1.227 | 0.684 |
| Health disability | 0.980 | 0.852 – 1.126 | 0.773 |
| Living environment | 0.914 | 0.814 – 1.027 | 0.131 |
| Medical history |  |  |  |
| Diabetes mellitus | 1.526 | 0.835 – 2.790 | 0.170 |
| Ischaemic heart disease | 1.398 | 0.638 – 3.062 | 0.403 |
| Patent foramen ovale | 0.636 | 0.287 – 1.409 | 0.265 |
| Migraine | 1.227 | 0.597 – 2.521 | 0.578 |
| Hypertension | 0.990 | 0.606- 1.617 | 0.968 |
| Stroke type |  |  |  |
| Ischaemic stroke | Ref | Ref | Ref |
| Intracerebral haemorrhage | 2.023 | 1.118 – 3.660 | ***0.020** |
| Admission NIHSS (per point) | 1.113 | 1.067 – 1.160 | ***<0.001** |
| Pre-morbid mRS |  |  |  |
| No disability (mRS 0-1) | Ref | Ref | Ref |
| Disability (mRS 2-5) | 3.171 | 1.100 – 9.140 | ***0.033** |

Variables with p-values <0.2 in the univariable analysis (Supplementary Table S4) were included in the multivariable model. Medication history, inpatient treatment, discharge mRS, and stroke recurrence were either omitted from the model or were excluded because they are not applicable to intracerebral haemorrhage patients in the cohort. Hypertension was included in the model as a potential confounding factor. * denotes statistically significant variables (*p*<0.05). mRS, modified Rankin Scale; NIHSS, National Institute of Health Stroke Scale; TIA, transient ischaemic attack; IQR, interquartile range.
